# Essential Oil Delivery Route: Effect on Broiler Chicken’s Growth Performance, Blood Biochemistry, Intestinal Morphology, Immune, and Antioxidant Status

**DOI:** 10.3390/ani11123386

**Published:** 2021-11-26

**Authors:** Samson Oladokun, Janice MacIsaac, Bruce Rathgeber, Deborah Adewole

**Affiliations:** Department of Animal Science and Aquaculture, Faculty of Agriculture, Dalhousie University, Truro, NS B2N 5E3, Canada; samson.oladokun@dal.ca (S.O.); janice.macisaac@dal.ca (J.M.); brathgeber@dal.ca (B.R.)

**Keywords:** in ovo, essential oil, blood biochemistry, antioxidant, broiler chicken

## Abstract

**Simple Summary:**

Much research is devoted to the search for potent alternatives to antibiotic growth promoters in the poultry industry. It is hypothesized that the efficacy of potential alternatives could be influenced by their type and the delivery strategy utilized. Consequently, this study evaluated the efficacy of a commercial essential oil blend across different delivery routes as a potent alternative to in-feed antibiotics in broiler chickens using selected biochemical, immune, and performance parameters. The results provide evidence that the successive delivery of essential oils via in ovo and in-water routes in broiler chickens offers the potential to improve broiler chicken biochemical and antioxidant status. However, the in ovo delivery of essential oil at 0.2 mL dosage (saline + essential oil, dilution ratio—2:1) suffers the limitations of reduced hatchability.

**Abstract:**

This study evaluated the effect of an essential oil blend and its delivery routes on broiler chicken growth performance, blood biochemistry, intestinal morphology, and immune and antioxidant status. Eggs were incubated and allotted to 3 groups: non-injected group, in ovo saline group, and in ovo essential oil group. On day 18 of incubation, essential oil in saline or saline alone was injected into the amnion. At hatch, chicks were assigned to post-hatch treatment combinations (1) in ovo essential oil + in-water essential oil (in ovo + in-water EO); (2) in ovo essential oil (in ovo EO); (3) in ovo saline; (4) in-water essential oil; (5) in-feed antibiotics (Bacitracin methylene disalicylate) and (6) a negative control (NC; corn-wheat-soybean diet) in 8 replicate cages (6 birds/cage) and raised for 28 day. The in ovo EO group reduced (*p* < 0.05) chick length and hatchability, all groups recorded no difference in growth performance at 0–28 day. The in ovo + in-water EO treatment reduced (*p* < 0.05) blood creatine kinase and aspartate aminotransferase levels whilst increasing (*p* < 0.05) total antioxidant capacity in birds. The in ovo + in-water delivery of EO might represent a potential antibiotic reduction strategy for the poultry industry but more research is needed to address the concern of reduced hatchability.

## 1. Introduction

The poultry meat industry is growing fast and is the cheapest source of animal protein for humans [1]. This substantive growth in the poultry industry has, over the years, been facilitated by the sub-therapeutic use of antibiotic growth promoters (AGPs) [2]. The supplementation of AGPs at sub-therapeutic levels is broadly used to improve the growth rate, feed efficiency, and reduce morbidity and mortality in poultry birds [3]. However, the continuous use of AGPs in the poultry industry has come under scrutiny due to public health concerns bordering on the emergence of antibiotic resistance [4]. Consequently, a few countries have instituted restrictions against the use of AGPs in the poultry industry. For example, the European Union (EU) banned AGPs as far back as 2006 [5]. The US Food and Drug Administration (FDA) also issued industry guidance on the prohibition of voluntary labeling of medically important animal drugs for animal growth promotion in 2013 [6]. Canadian poultry producers eliminated the preventive use of category 1 and 2 antibiotics in 2014 and 2018, respectively [7], while China also banned the use of AGPS in 2020 [8]. To preserve the potency of medically important antibiotics for human use, prevent the emergence of public health risks associated with the use of AGPs, satisfy increased consumer demands for antibiotic-free poultry products, and to sustain increased poultry production trends, there is a dire need for the development of safe, cost-effective, eco-friendly, and effective alternatives to AGPs for the poultry industry.

Several bioactive substances are being evaluated as potential alternatives to AGPs as reviewed by Gadde et al. [9]. These bioactive substances include probiotics, prebiotics, symbiotics, organic acids, enzymes, and several phytogenic feed additives (PFAs). The solid, dried, ground form or extracts from plants constitute these PFAs. Based on the extraction procedures, PFAs can be broadly classified as oleoresins (extracts derived by non-aqueous solvents) and essential oils (EOs; extracts obtained by cold, steam, or alcohol distillation) [10,11]. Although the major component of most EOs, such as thymol, carvacrol, and eugenol, are phenolic compounds (terpenoids and phenylpropanoids) [12], EOs vary in individual chemical compositions and concentrations. For example, as low as 3% and as high as 60%, thymol and carvacrol have been reported as the total EO in thyme [13] and a cinnamaldehyde range of 60% to 75% in cinnamon EOs [14]. The activity of EOs is strongly associated with their chemical composition, functional groups, and synergistic interactions between components [15,16]. Common aromatic oils utilized in poultry production include oils from garlic (*Allium sativum*), oregano (*Origanum vulgare*) turmeric, (*Curcuma longa*), lemon balm (*Melissa officinalis*), peppermint (*Mentha piperita*), star anise fruit (*Illicium verum*), cinnamon (*Cinnamomum zeylanicum*), rosemary (*Rosmarinus officinalis*), and thyme (*Thymus vulgaris*) [17,18,19,20,21]. To explore a synergistic effect, commercial combinations, or a blend of several EO types is becoming increasingly popular. Across the literature, several in vitro studies have highlighted the antibacterial, antiviral, antifungal, antimycotic, antiparasitic, insecticidal, antioxidant, anti-inflammatory, anti-toxigenic, anti-quorum-sensing, and immune-regulating properties of EOs [22,23,24,25]. Contrastingly, in vivo results reported in the literature on the effect of EOs on poultry performance are somewhat inconsistent. While a few studies have reported the positive effect of EOs on poultry performance, digestive function, immune response, antioxidant capacity, and meat quality [26,27,28], other studies have equally recorded poor [29] or no effect of EOs on poultry production parameters [30,31,32,33,34,35,36,37]. These inconsistencies in the efficacy of EOs have been associated with the limitations that characterize their mode of delivery [38,39], as most EOs are conventionally supplied via feed or water to poultry birds. These conventional routes limit the efficacy of EOs because EOs are extremely volatile, easily degradable, and sensitive to environmental variables [3,39]. For example, when supplied in the diet, pelleting temperature of 58 °C have been reported to cause considerable loss of EO activity [40]. Additionally, EOs may potentially interact with the composition of basal diets, hence limiting their efficacy [41,42,43]. On the other hand, when supplied via the in-water route, the efficacy of EOs will depend on the quality of the water, and the quality of chick watering devices.

To overcome the identified challenges that characterize conventional delivery routes and, by extension, the efficacy of EOs; in ovo delivery has been proposed. In ovo technology has been defined as “the direct inoculation of bioactive substances to the developing embryo to elicit superior lifelong effects, while considering the dynamic physiology of the chicken embryo” [44]. This mode of delivery offers a few advantages over conventional delivery routes. These advantages include an economic benefit, as fewer bioactive substances are reportedly needed to elicit similar performance-enhancing effects as conventional routes [45,46]. Additionally, in ovo delivery also offers scope for early immunomodulatory programming and nutritional intervention in chicks [44]. Interestingly, research on the in ovo delivery of EOs in poultry is relatively scarce in the literature.

Accordingly, the objective of this study was to evaluate the effect of the in ovo delivery of a commercial EO blend (containing star anise, cinnamon, rosemary, and thyme oil) on hatch and growth performance, immune and antioxidant status, blood biochemistry, and intestinal morphometric properties in broiler chickens, compared to conventional delivery routes. This is the first study evaluating the efficacy of in ovo delivered EOs compared to AGPs within the limits of the available literature. This study also sought to evaluate if an additive benefit exists from the successive delivery of EOs via the in ovo and continuous in-water delivery routes. From available knowledge, this is also the first study seeking to evaluate such an effect.

## 2. Materials and Methods

The experiment was carried out at the hatchery facility of the Agricultural Campus of Dalhousie University and the broiler rearing facility of the Atlantic Poultry Research Center, Dalhousie Faculty of Agriculture. All experimental procedures were approved by the Animal Care and Use Committee of Dalhousie University (Protocol number: 2020-035), following guidelines of the Canadian Council on Animal Care [47].

### 2.1. Egg Incubation and In Ovo Injection Procedure

A total of 670 hatching eggs with an average weight of 77.87 ± 2.43 g (mean ± SE) from 41-week-old Cobb 500 broiler breeders were sourced from a commercial hatchery (Synergy hatchery) in Nova Scotia, Canada. Eggs were incubated in a ChickMaster single-stage incubator (ChickMaster G09, Cresskill, NJ, USA) under standard conditions (37.5 °C and 55% relative humidity) from embryonic days (EDs) 1 to 17, and then to an average of 32 °C and 68% from EDs 18 to 21. Incubators were preheated for 24 h prior to setting eggs to ensure that proper temperature and humidity were stable. Egg trays were turned on a 90° arc four times an hour from the time of set until ED 18. Eggs were arranged in 6 replicate trays inside the incubator, each tray containing 96 eggs. On ED 12, eggs were candled, and infertile eggs were disposed of, leaving a total of 576 eggs for the trial. The remaining eggs were subsequently assigned to one of three treatment groups: (1) non-injected eggs (control; 288 eggs); (2) in ovo saline group (96 eggs; injected with 0.2 mL of physiological saline, i.e., 0.9% NaCl); (3) in ovo essential oil group (192 eggs; injected with 0.2 mL of a saline + essential oil blend mixture at a dilution ratio of 2:1). The essential oil utilized in this study is a commercial blend (Probiotech International Inc., St Hyacinthe, Quebec, Canada) containing phytonutrients star anise, cinnamon, rosemary, and thyme oil. The EO blend is registered by Health Canada as a Veterinary Health Product (VHP). On ED 18, eggs were injected according to the procedure described by Oladokun et al. [48], with slight modifications. Briefly, this involved disinfecting the eggs with 70% ethanol-dipped swabs and using an 18-gauge needle to carefully punch the shell at the center of the air cell (the blunt end). The injected EO was then delivered to the amnion using a self-refilling injector (Socorex ultra-1810.2.05005, Ecublens, Switzerland) equipped with a 22-gauge needle (injection needle length—3 cm) at a 45-degree angle. After in ovo injection, the injection sites were sealed with sterile paraffin, and eggs were placed back in the incubator. The non-injected eggs were also taken out and returned to the incubator simultaneously as other injected treatment groups.

### 2.2. Birds, Housing, and Diets

**Figure 1:**
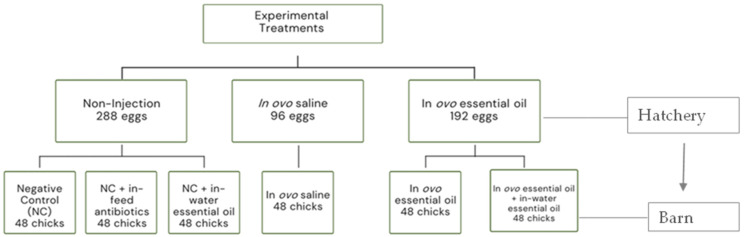
Schematic presentation of experimental structure in the hatchery and barn.

As presented in Figure 1, hatchlings were weighed and randomly assigned to 6 new treatment groups. Chicks from the initial non-injection group were randomly allocated into four new treatment groups consisting of (1) chicks fed a basal corn-soybean meal-wheat–based diet (Negative Control treatment; NC); (2) chicks fed NC + 0.05% bacitracin methylene disalicylate (in-feed antibiotics); and (3) chicks supplied the same commercial blend of EOs as earlier described via the water route (in-water essential oil) at the recommended dosage of 250 mL/1000 L of drinking water. The initial in ovo saline and in ovo essential oil groups were placed on the control diet to form treatments (4) (in ovo saline treatment) and (5) (in ovo essential oil treatment), respectively. The last treatment group, (6) consisted of chicks from the in ovo essential oil treatment group also supplied EO via the water route (in ovo + in-water essential oil treatment). All treatment groups had 48 birds each. Birds were placed in battery cages (0.93 m × 2.14 m), there were 6 birds per cage and 8 replicate cages per treatment. To minimize variability, only the top top-tier cages were used; each treatment group was evenly represented across a tier. Birds were reared for 28 day under uniform controlled environmental conditions in line with Cobb Broiler Management Guide recommendations. Room temperature was set at 31 °C on day 0 and gradually reduced to 23 °C on day 28, and relative humidity ranged between 45 and 55%. Dietary treatments, ingredients, and nutritional composition are presented in Table 1. Birds were provided with feed and water ad libitum; diets were fed as mash throughout the rearing period, including the starter (0–14 d) and grower (15–28 d) phases. Diets met or exceeded the NRC [49] nutritional requirements for broiler chickens.

### 2.3. Hatch Parameters and Chick Quality

Hatched chicks were counted and weighed individually. Hatchability was calculated as the percentage of hatched chicks to fertile incubated eggs per replicate. Hatched chick BW/initial egg weight ratio was also determined and recorded. Chick navel quality was evaluated by adopting the scoring method by Reijrink et al. [50]. Navel quality was scored 1—when the navel was completely closed and clean; scored 2—when the navel was discolored (i.e., when the navel color differs from the chick’s skin color) with a maximum 2 mm opening; and scored 3—when the navel was discolored and with more than a 2 mm opening. Chick length was obtained by placing the chick on its ventral side and measuring from the tip of the beak to the middle toe on the right leg.

### 2.4. Growth Performance Parameters and Sampling

Growth performance parameters—feed intake and average body weight (BW) were measured on a cage basis weekly. The average feed intake (AFI), average body weight gain (ABWG), and feed conversion ratio (FCR) were subsequently calculated from the obtained data. The FCR was calculated as the amount of feed consumed per unit of body weight gain. Cages were checked for mortality daily; dead birds were subsequently weighed and sent to the Nova Scotia Agriculture, Animal Health Laboratory for necropsy. Mortality weight was then used to correct the FCR.

On day 21, 1 bird per cage (8 replicate birds per treatment group) was randomly selected, weighed, and euthanized by electrical stunning and exsanguination. After euthanasia of the bird, blood samples were collected from each bird into 10 mL blood serum collection tubes (BD Vacutainer™ Serum Tubes, fisher scientific- BD366430) for serum immunoglobulins assay. After slaughter, the weights of the bursa of Fabricius and liver were also determined by trained personnel.

On day 28, 2 birds per cage (16 replicate birds per treatment group) were randomly selected and euthanized by electrical stunning and exsanguination. After euthanasia of the bird, blood samples were collected from each bird into 10 mL heparinized tubes (BD Vacutainer™ Glass Blood Collection Tubes with Sodium Heparin, fisher scientific- BD366480) for blood biochemistry and plasma total antioxidant assays. After slaughter, the small intestinal segments, including the jejunum (1.5-cm length midway between the point of entry of the bile ducts and Meckel’s diverticulum) and ileum (1.5-cm length midway between Meckel’s diverticulum and the ileocecal junction) were excised and fixed in neutral buffered formalin (10%) for further histomorphological processing [51].

#### 2.4.1. Relative Weight of Organs

The weights of the bursa of Fabricius and liver were recorded and then specified as a percentage of the live BW of the slaughtered chicken (g/Kg BW).

#### 2.4.2. Serum Immunoglobulins

Serum samples were used to measure concentrations of immunoglobulins (IgG and IgM) using chicken-specific immunoglobulins enzyme-link immunosorbent assay (ELISA) quantitation kits (Bethyl Laboratories, Montgomery, TX, USA; Catalog No. E33-104-200218 and E33-102-180410, respectively) following manufacturer instructions. The values were determined on a microplate reader (Bio-Tek Instrument Inc., Wonooski, VT, USA) using a software program (KC4, version #3.3, Bio Tek Instruments). The four-parameter logistic model was used to extrapolate immunoglobulins concentration and absorbance readings.

#### 2.4.3. Blood Biochemistry

Samples for blood biochemical analysis were centrifuged at 5000× *g* at 4 °C for 10 min and shipped on ice to Atlantic Veterinary College, University of Prince Edward Island Pathology Laboratory, where samples were analyzed using cobas^®^ 6000 analyzer series (Roche Diagnostics, Indianapolis, IN, USA).

#### 2.4.4. Total Antioxidant Capacity (TAC)

Total antioxidant capacity (TAC) in plasma was analyzed using the Oxiselect Total Antioxidant Capacity assay kit (STA360; Cell BioLabs Inc., San Diego, CA, USA) following the manufacturer’s instructions [52]. Absorbance was measured at 490 nm on a microplate reader (Bio-Tek Instrument Inc., Wonooski, VT, USA) using a software program (KC4, version #3.3, Bio Tek Instruments). Results were expressed as mM Uric acid equivalent.

#### 2.4.5. Intestinal Morphology

The procedure for intestinal morphometric analysis was as described by Oladokun et al. [48]. Briefly, fixed intestinal tissues were embedded in paraffin, sectioned (0.5 μm thick), and stained with hematoxylin and eosin for morphological examinations. In each cross-sectioned tissue, ten morphometric measurements including the villus height (from the base of the intestinal mucosa to the tip of the villus excluding the intestinal crypt), villus width (halfway between the base and the tip), crypt depth (from the base upward to the region of transition between the crypt and villi) [53] per slide were carried out using Leica 1CC50 W microscope at 4× Magnification (Leica Microsystems, Wetzlay, Germany) and an image processing and analysis system (Leica Application Suite, Version 3.4.0, Leica Microsystems, Wetzlay, Germany). The total mucosa thickness (villus height + crypt depth) was subsequently calculated from the obtained data.

### 2.5. Statistical Analysis

Hatch data were analyzed as a completely randomized design, while other datasets obtained were analyzed as a randomized complete block design, with cage-tiers being the blocking factor. The normality of all data sets was ascertained by testing residuals by the Anderson-Darling test in Minitab statistical package (v.18.1). Data sets found to be normal were subjected to one-way ANOVA in the same statistical package with experimental treatments as a factor and the relevant data sets as variables. Data sets found not to be normal, including plasma protein, globulin, and bile acids, were transformed using the reciprocal function. Data on plasma potassium, chloride, and magnesium were transformed using the reciprocal cube function, while plasma glucose and chloride were transformed by the square reciprocal function. Data on plasma alkaline phosphatase (ALP), uric acid, serum IgG were transformed using the natural log function. Data on plasma aspartate aminotransferase (AST), creatine kinase (CK), and urea were transformed using the logarithm base ten functions. Following data transformations, the transformed data were equally subjected to ANOVA procedures in the same statistical package, with appropriately back-transformed data presented. For hatchability parameters, hatching trays were the experimental units, and the pen was the experimental unit for growth performance parameters. Significant means were separated using Tukey’s honest significant difference test in the same statistical package. Analyzed data were presented as means ± SEM and probability values. Values were considered statistically different at *p* ≤ 0.05 and considered a statistical trend at *p* < 0.1.

## 3. Results

### 3.1. Hatch Performance and Chick Quality

The results on hatch performance and chick quality are presented in Table 2. No effect of treatment was recorded for the average chick weight and average navel score parameters. However, both hatchability and average chick length were significantly (*p* < 0.05) affected by treatments. The in ovo essential oil treatment recorded an 18.1 and 19.5% reduction in hatchability compared to the non-injected and in ovo saline treatment, respectively. Similarly, the in ovo essential oil treatment also recorded a 3.7 and 3.2% reduction in average chick length compared to the non-injected and in ovo saline treatment, respectively.

### 3.2. Growth Performance

Results on evaluated growth parameters are presented in Table 3. At the starter phase (d 0–14), antibiotic treatment recorded higher (*p* < 0.05) ABWG compared to all in ovo-delivered treatments (in ovo saline, in ovo EO, in ovo + in water EO). At the grower phase (d 15–28) and for the entire length of study (d 0–28), there was no treatment effect on evaluated growth performance parameters.

### 3.3. Relative Weight of Organs and Serum Immunoglobulins

Treatments recorded no significant effect on serum IgG and IgM levels in broiler chickens in this study (Table 4). Results on evaluated organ weights are also presented in Table 4. The relative weight of the bursa was equally not affected by treatments in this study. However, the in ovo + in-water essential oil treatment recorded a tendency (*p* = 0.07) to increase the relative weight of the liver compared to other treatments. The relative liver weight of birds in this treatment was 15% heavier than the NC treatment and at least 6% heavier than other treatments.

### 3.4. Blood Biochemistry

The effects of treatments on blood plasma biochemical characteristics are presented in Table 5. Blood enzymes—CK and AST, were significantly affected by treatments. In ovo saline and in ovo + in-water essential oil treatments, both significantly reduced (*p* < 0.05) plasma CK levels, compared to the in-feed antibiotics treatment. Nonetheless, the highest reduction in plasma CK levels was observed in the in ovo + in-water essential oil treatment; this was as much as about a 3-fold reduction, compared to the in-feed antibiotics treatment. The NC, in-water, and in ovo essential oil treatments recorded intermediate plasma CK levels. Compared to the NC and in-feed antibiotics treatments, blood plasma AST levels were significantly reduced (*p* < 0.05) by the in-water, in ovo saline, and in ovo + in-water essential oil treatments. Nevertheless, the in ovo + in-water essential oil treatment recorded the highest reduction in plasma AST level, 29.6% lower than the in-feed antibiotics treatment. Furthermore, the in ovo + in-water essential oil treatment recorded a tendency (*p* = 0.07) to increase plasma calcium level by as much as 12.6%, relative to the NC treatment. All other evaluated blood plasma characteristics evaluated in this study were not affected by treatments.

### 3.5. Total Antioxidant Capacity (TAC)

**Figure 2:**
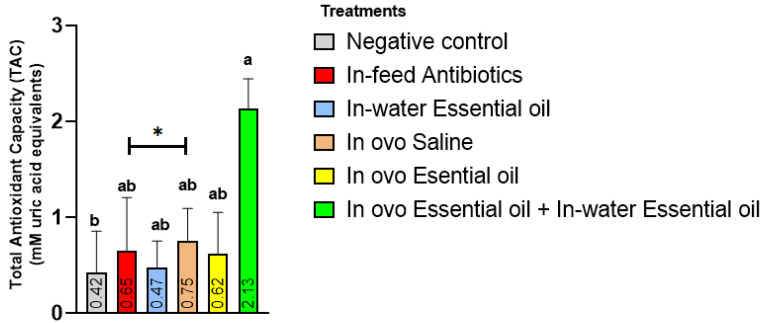
Effect of essential oil delivery route on broiler chicken’s total antioxidant capacity (TAC). Bar charts with different letters ^a, b^ differ (* indicates a significant difference at *p* < 0.05).

The result on TAC is presented in Figure 2. The in ovo + in-water essential oil treatment significantly increased (*p* < 0.05) TAC in birds compared to the NC treatment. This increase in TAC in the in ovo + in-water essential oil treatment was as much as 5-fold the NC treatment. Other treatments recorded intermediate TAC values.

### 3.6. Intestinal Morphology

The morphology of the duodenum and ileum was significantly influenced by treatments in this study (Table 6). No effect of treatment on jejunum morphology was found. In the duodenum, the in ovo essential oil treatment recorded the longest (*p* < 0.001) villus compared to other treatments, except for the in ovo + in-water essential oil treatment. The in ovo + in-water essential oil treatment recorded intermediate duodenal villus length. The duodenal villus width of birds in the in ovo treatment group was only wider (*p* = 0.01) than those in the in ovo saline treatment group; all other treatments recorded intermediate duodenal villus width. Similarly, total mucosa thickness was also highest (*p* < 0.001) in the in ovo essential oil treatment compared to the in-feed antibiotic, in-water and in ovo saline treatments. In the ileum, the in ovo essential oil treatment and the NC treatment recorded significantly longer (*p* < 0.001) villus height than the in-water essential treatment. Other treatments recorded intermediate villus height values. Similarly, total mucosa thickness in the ileum was significantly enhanced (*p* < 0.001) by the in ovo essential oil treatment compared to the in-water essential oil treatment. Statistical intermediate total mucosa thickness was recorded for other treatments in the ileum.

## 4. Discussion

As the search for effective alternatives to AGPs for the poultry industry continues, there is also a need for the urgent development of delivery strategies that optimize their effectiveness. The potential of several EOs extracted from herbs and spices as alternatives to AGPs continues to be recognized due to their biological properties. For instance, EOs derivable from the star anise plant has been reported to have growth-promoting [54], antioxygenic [55], antibacterial, and digestion-enhancing properties [56]. Similarly, cinnamon EO has cinnamaldehyde (3- phenyl-2-propenal) as its major component, conferring it antimicrobial [57], cardio-protective [58], antidiabetic [59], and hypocholesterolemic properties [60]. Rosemary extract has carnosic acid, carnosol, rosmanol, rosmariquinone and rosmaridiphenol, ursolic acid, and caffeic acid as major phenolic components [61]. The antimicrobial, anti-inflammatory, antidiabetic, anticancer, and antioxidant properties of rosemary EOs have also been documented [62,63,64,65,66,67,68,69,70]. Thyme EO also has thymol, carvacrol, and linalool as its main active compounds [35,66]. The antimicrobial, antioxidant, and digestion-enhancing properties of thyme EO are also well documented [67,68,69]. Despite the beneficial biological properties that each of these EO can exhibit, an accurate blend of these EOs can manifest greater responses via a synergistic mode of action [70,71,72]. Accordingly, the first comparison of an essential oil blend with an AGP across several delivery routes in the literature is thus presented herein.

In this study, in ovo delivery of EOs reduced hatchability and chick length in broiler chickens. Conversely, most of the very limited studies on in ovo delivered EOs have recorded no effect [73,74] or increased hatchability [75,76]. Compared to this study, the differences in injected EO nature, concentration, volume, injection site, and dosage might potentially explain the observed result. These factors have previously been highlighted as critical to the success of in ovo delivery [44]. The impaired hatchability recorded by the in ovo delivery route at this injection dosage (0.2 mL of a saline + essential oil blend mixture at a dilution ratio of 2:1) is considered a significant limitation that could prevent the adoption of this delivery route for EO delivery by poultry producers. More research is thus needed to optimize standard guidelines regarding these factors in order to guarantee successful in ovo delivery of EOs. In principle, the antioxidant capacity of injected EOs is expected to mitigate the overproduction of free radicals, and in consequence, cause increased hatchability [76,77,78]. Chick length is often used to predict chick growth potential [79]. As this is the first study on in ovo delivery of EOs to evaluate this parameter, there was no basis for comparison with other studies. Thus, it can only be speculated that the in ovo delivered EOs dose was unfavorable to the hatched chicks. Ideally, in ovo delivered EOs after day 18 are expected not to influence chick length. This is because any biological substances delivered to the embryo after day 18 are devoted to providing energy for the hatching process and not for organogenesis; in ovo delivery of nutrients is the perfect candidate for this time-point. On the other hand, biological substances provided to the embryo before the first 18 days of incubation are devoted to embryo organogenesis and growth [80,81]. It is also important to state that the commercial EO blend utilized in this study is not dedicated to being in ovo administered, per default, and contained emulsifiers as prepared for in-water administration. Therefore, bioactive substances intended for in ovo administration might require specific formulation. As the concept of in ovo delivery of bioactive substances other than vaccines in poultry is relatively new, a need for specially formulated commercially available bioactive substances thus exists. Furthermore, besides injection dosage, egg source and egg quality might also potentially impair hatch performance and chick quality. Following in ovo delivery of EO + saline, increased chick weight and no effect on chick length were recorded during the pretrial stage of this study (Supplementary Materials Table S1).

At the end of the trial (d 28), all treatments recorded no significant effect on the evaluated post-hatch growth performance parameters (AFI, ABWG, and FCR) in this study. Only in the starter phase (d 0–14) did the antibiotic treatment record a significant increase in ABWG. The growth-enhancing properties of AGPs are well substantiated across the literature [5,9,82]. Generally, the effect of EOs on bird performance is observed to be inconsistent across the literature. While the result presented here are consistent with other studies that have reported no effect of EOs on bird post-hatch growth performance [20,32,33,35,36,37,83,84,85,86,87,88,89,90,91,92,93]. Contrastingly, other studies have recorded increased [75,92,93,94] and decreased growth performance on EO supplementation [95]. The variability in the growth performance effect of EOs is attributable to several intrinsic and extrinsic factors that include the physiological status of the bird, housing type (cage vs. floor pens), housing hygiene (clean vs. unclean) as challenge acuity index, basal diet composition, and time of rearing [11,32,93,96,97]. It would be reasonable to speculate that the short timing of this study (28 days) and housing type (battery cages) largely influenced the obtained results on growth performance. Moreover, digestive enzyme secretion capacity is reportedly limited in young chicks [98], while nutrient requirements decrease with increasing age [49]. Additionally, due to a well-developed digestive tract and organs, older birds are thus able to utilize the finisher diets better [99].

Immunoglobulins are synthesized by the B cells to regulate humoral immunity. They are often synthesized in response to immune stressors such as infection and oxidative stress [100]. This study recorded no effect of EOs and their delivery routes on serum immunoglobulins G and M levels. This result was not surprising as birds were raised under experimental and controlled conditions with no strain on their immune system. Previous studies have also reported no effect of EOs on the level of immunoglobulins G and M in broiler chickens [101,102,103], laying hens [104], and rabbits [105]. While a few studies have recorded increased immunoglobulins G and M levels following EO supplementation [75,76,84], Movahhedkhah et al. [106] have suggested an age-dependent immune response on EOs supplementation exists in birds. The authors suggested evaluating this parameter at the later stage of growth in broiler chickens. In any case, the exact mechanism by which EOs stimulate an immunological response in birds is not fully known, thus warranting further investigation. Furthermore, a tendency for increased relative weight of the liver in the in ovo + in-water EO treatment was observed in this study. An increase in the relative weight of the liver in birds receiving thymol [35], oregano oil [107], EO blend containing thyme and cinnamon [108], and EO blend containing oregano and sage oil [109] have been reported. The observed tendency for increased liver weight exerted by the in ovo + in-water EO treatment could be attributed to decreased apoptosis of liver tissues resulting from the antioxidant properties of the delivered EO blend [108].

Blood biochemistry indices are valuable indicators of the health and wellbeing of the bird. The plasma biochemistry indices observed in this study were all within the normal physiological range for broiler chickens [96,110,111]. The highest reduction in the level of blood enzyme CK was observed in the in ovo + in-water EO treatment in this study. The blood enzyme CK is an intracellular enzyme whose plasma concentration is usually used as an indicator of skeletal muscle damage [112]. Skeletal muscle damage is inducible by congenital myopathies, nutritional myopathies, or oxidative stress. In agreement with the result presented here, Salehifar et al. [113] have reported the efficacy of lemon pulp powder to decrease the activities of CK in heat-stressed broilers. Similarly, EO blends containing rosemary oil [114] and *Curcuma xanthorrhiza* oil [115] have all been reported to reduce CK levels in broiler chickens significantly. The reduction in blood CK levels could be associated with the antioxidant capacities of the delivered EO blends, which was enhanced by the in ovo + in-water EO delivery route. This same delivery route (in ovo + in-water EO) also recorded the highest reduction in plasma AST level. Increased levels of AST in the blood are often indicative of increased permeability of liver cells and liver damage [116,117]. Consistent with the results from this study in ovo delivered cinnamon, thyme, and clove oil have all been reported to individually reduce serum AST levels in broiler chickens [92]. Several supplemented EOs have equally reported similar AST lowering effects [116,118,119,120]. The hepatoprotective effect provided by the in ovo + in-water EO delivery route is possibly due to the rich antioxidant compounds present in the supplemented EO blend. Additionally, this hepatoprotective effect is also effectuated by the induction of endogenous interferon [121]. A tendency for increased plasma calcium level was also recorded in the in ovo + in-water EO delivery route. Blends of EO containing thyme, black cumin, fennel, anise, and rosemary have been reported to increase the calcium concentration in the tibia of laying hens [122]. The same EO blend has also been reported to decrease calcium excretion in breeder quails [91]. Ileal calcium bioavailability has also been enhanced by the supplementation of EO in broilers [43,123]. This tendency for increased plasma calcium level in the in ovo + in-water EO delivery route is possibly induced by increased mobilization of calcium-binding protein in the mucosa, activating the calcium-activated tenderization complex. This observation has implications for improving bone strength and bird leg health [124].

An apparent increase in TAC was also observed in the in ovo + in-water EO treatment, buttressing that this delivery route clearly enhanced the antioxidant potential of the delivered EO blend. The values of TAC are indicative of the overall antioxidant defense systems, both enzymatic and non-enzymatic. Several in vitro studies have reported the antioxidant properties of several plant extracts and EOs [125,126,127]. Increased levels of TAC resulting from supplementation of star anise EO in laying hens [128] and broilers [129], oregano powder in broiler chickens [130], oregano EO in broiler chickens [131], *Satureja officinalis* EO in broiler chickens [132], and rosemary EO in rabbits [105] have been reported. Varying antioxidant capacities are reported for most evaluated EOs in the literature, justifying the need for research evaluating various EO combination types and delivery routes in order to ensure EO efficacy either by synergistic or additive mechanisms. For instance, rosemary is regarded as the plant with the highest antioxidant capacity [133], while the antioxidant capacity of oregano EO is also reported to be greater than vitamin E [134]. Thymol is also reported to exhibit greater antioxidant capacity than carvacrol, possibly because thymol has greater stearic inhibition of the phenolic group than carvacrol [135]. The antioxidant properties of these EOs are due to the presence of phenolic OH groups in their chemical structure; this acts as a hydrogen donor interacting with peroxyl radicals during the initial process of lipid oxidation and thereby inhibiting the formation of hydroxy peroxide [35]. Another potential mode of action that requires further research is via the upregulation of antioxidant-related genes.

It is well documented that the structure and morphology of a bird’s small intestine influence its functionality. The in ovo EO delivery route enhanced duodenal and ileal morphology in this study. While the villus length, width, and total mucosa thickness were enhanced by in ovo delivery of this treatment in the duodenum, only the villus height and total mucosa thickness were enhanced by this delivery route in the ileum. The duodenum is an important site for chemical digestion, while the ileum plays a vital role in starch digestion and absorption, especially in fast-growing broiler chickens [136]. Increased villus height, villus width, and total mucosa thickness are generally associated with improved digestive and absorptive functions in the bird [137,138]. To our knowledge, this is the first study to evaluate the effect of in ovo delivered EOs on broiler chicken’s intestinal morphology, thus providing limited scope to compare obtained results. Nonetheless, several aromatic plants and their extracts are reported to enhance the intestinal morphology of broiler chickens [33,139]. A blend of EOs containing star anise and oregano oil is also reported to increase the height of duodenal villi in broiler chickens [140]. Similarly, dietary supplementation of 300 mg cinnamon bark oil kg–1 also reportedly increased villus height in the duodenum and ileum of broiler chickens [141]. Blend of EOs containing basil, caraway, laurel, lemon, oregano, sage, tea, and thyme is also reported to significantly increase the width and surface area of the small intestine [96]. Similarly, both [142,143] have also reported increased villus length with in-water EO supplementation in quails and broiler chickens, respectively. The beneficial effect of the in ovo EO route on intestinal morphology in this study might be attributed to the early time of delivery. Moreover, the development of the small intestine in chicks has been described to be synonymous with the mammalian neonates, with the greatest morphological change occurring within the first 24 h post-hatch. The in ovo technology has been recognized to be a means to stimulate the development of the embryonic gastrointestinal tract (GIT) [44]. The potential of the active ingredients in the EO blend to stimulate the secretion of endogenous digestive enzymes while also ensuring a balanced gut microbial diversity could also have contributed to the observed effect [144,145,146]. The antimicrobial, anti-inflammatory, and antioxidant properties of the supplied EO blend also play an important role in gut morphometric development [146,147]. Improved intestinal digestion and absorption due to improved intestinal morphology facilitated by the in ovo delivery route would be expected to translate into improved growth performance in the birds. However, this was not the case in this study. It has been previously speculated that the length of this study and the housing type could have contributed to the observed results on growth performance.

## 5. Conclusions

This study revealed that the in ovo delivery of EO blends containing star anise, cinnamon, rosemary, and thyme oil reduced hatchability and chick length in broiler chickens. However, successive delivery of this EO blend via in ovo and in-water route improved broiler chicken’s antioxidant status and blood biochemical profile, with no adverse effect on growth performance. Additionally, in ovo delivery of this EO blend also improved intestinal morphometric properties of the bird. Based on observed hatchability and chick length results, it would be essential to optimize injected EO dose through further studies. Furthermore, considering that the supplied EO blend has reported antioxidant and immune-enhancing properties, it would be interesting to evaluate the efficacy of the in ovo + in-water EO delivery route under a heat stress challenge model. Heat stress could potentially induce oxidative stress and immunosuppression in birds. Conclusively, subject to further research with favorable hatchability outcomes, this novel delivery strategy might be a potential alternative to the use of antibiotics in the poultry industry.

## Figures and Tables

**Table 1:** Ingredients, calculated, and analyzed compositions of experimental diets ^1^ (as-fed basis, percentage (%), unless otherwise stated).

Ingredients	Phases			
	Starter (0–14 d)		Grower (15–28 d)	
	Control Diet	Antibiotic Diet	Control Diet	Antibiotic Diet
Ingredient Composition				
Corn (ground)	51.08	50.98	45.36	45.25
Soybean meal-46.5	41.44	41.45	36.31	36.33
Wheat	-	-	10	10
Animal/vegetable fat	2.93	2.97	4.22	4.26
Limestone	1.80	1.80	1.65	1.65
Dicalcium Phosphate	1.24	1.24	1.06	1.06
DL Methionine premix ^2^	0.59	0.59	0.53	0.53
Vitamin/Mineral Premix ^3,4^	0.50	0.50	0.50	0.50
Salt	0.41	0.41	0.37	0.37
Lysine HCl	0.01	0.01	0.00	0.00
BMD 110G ^5^	-	0.05	-	0.05
Total	100	100	100	100
Nutrient	Calculated composition			
Metabolizable energy (kcal/kg)	3000	3000	3100	3100
Crude protein	23	23	21.5	21.5
Calcium	0.96	0.96	0.87	0.87
Available phosphorus	0.48	0.48	0.44	0.44
Sodium	0.19	0.19	0.18	0.18
Digestible lysine	1.28	1.28	1.15	1.16
Digestible methionine + cysteine	0.95	0.95	0.87	0.87
Digestible Tryptophan	0.25	0.25	0.23	0.23
Digestible Threonine	0.89	0.89	0.82	0.82
Analyzed composition				
Dry Matter	90.7	90.8	93.2	93.5
Crude protein	24.8	25	22.5	23.8
Crude fat	5.50	5.79	6.84	6.85
Calcium	1.06	1.13	1.00	0.96
Potassium	1.14	1.16	0.99	1.04
Phosphorus	0.69	0.70	0.67	0.62
Sodium	0.19	0.21	0.21	0.16

^1^ Basal diet (NC); antibiotic diet containing NC + 0.05% bacitracin methylene disalicylate (BMD). ^2^ Supplied/kg premix: DL-Methionine, 0.5 kg; wheat middling, 0.5 kg. ^3^ Starter vitamin-mineral premix contained the following per kg of diet: 9750 IU vitamin A; 2000 IU vitamin D3; 25 IU vitamin E; 2.97 mg vitamin K; 7.6 mg riboflavin; 13.5 mg Dl Ca-pantothenate; 0.012 mg vitamin B12; 29.7 mg niacin; 1.0 mg folic acid, 801 mg choline; 0.3 mg biotin; 4.9 mg pyridoxine; 2.9 mg thiamine; 70.2 mg manganese; 80.0 mg zinc; 25 mg copper; 0.15 mg selenium; 50 mg ethoxyquin; 1543 mg wheat middling’s; 500 mg ground limestone. ^4^ Grower and Finisher vitamin-mineral premix contained the following per kg of diet: 9750 IU vitamin A; 2000 IU vitamin D3; 25 IU vitamin E; 2.97 mg vitamin K; 7.6 mg riboflavin; 13.5 mg Dl Ca-pantothenate; 0.012 mg vitamin B12; 29.7 mg niacin; 1.0 mg folic acid, 801 mg choline; 0.3 mg biotin; 4.9 mg pyridoxine; 2.9 mg thiamine; 70.2 mg manganese; 80.0 mg zinc; 25 mg copper; 0.15 mg selenium; 50 mg ethoxyquin; 1543 mg wheat middling’s; 500 mg ground limestone. ^5^ Bacitracin methylene disalicylate (providing 55 mg/kg mixed feed); Alpharma, Inc., Fort Lee, NJ, USA.

**Table 2:** Effect of essential oil delivery route on hatch performance and chick quality.

Hatch Parameters	Treatments ^1^			SEM ^2^	*p* Value
	Non-Injected	In Ovo Saline	In Ovo Essential Oil		
Hatchability (%)	95.3 ^a^	97.0 ^a^	78.1 ^b^	4.10	0.001
Average Chick Weight (g)	52.3	49.7	47.4	2.00	0.590
Average Chick Length (cm)	18.9 ^a^	18.8 ^a^	18.2 ^b^	0.11	0.002
Chick BW/ Egg Weight (%)	56.7	70.2	64.4	3.00	0.183
Average Navel Score	1.67	1.51	1.77	0.10	0.299

^1^ Treatments include—(1) non-injected eggs; (2) in ovo Saline group- injected with 0.2 mL of physiological saline (0.9% NaCl); (3) in ovo essential oil group- injected with 0.2 mL of a saline + essential oil blend mixture at a dilution ratio of 2:1. ^2^ SEM = Standard error of means. ^a,b^ Means within a row with different superscripts differ (*p* < 0.05).

**Table 3:** Effect of essential oil delivery route on broiler chicken growth performance.

Growth Performance Parameters	Treatments ^1^						SEM ^2^	*p* Value ^3^
	Negative Control	In-Feed Antibiotics	In-Water Essential Oil	In Ovo Saline	In Ovo Essential Oil	In Ovo Essential Oil + In-Water Essential oil		
Average Feed Intake (g/bird)								
D 1–14	305	292	308	272	296	294	27.5	0.845
D 15–28	1297	1303	1497	1182	1166	1139	67.6	0.386
D 1–28	1599	1604	1790	1447	1461	1439	92.7	0.449
Average Body weight gain (g/bird)								
D 1–14	294 ^a,b^	307 ^a^	287 ^a,b,c^	246 ^c^	253 ^b,c^	250 ^b,c^	7.68	0.040
D 15–28	950	994	931	869	852	857	26.7	0.345
D 1–28	1243	1301	1217	1110	1104	1104	32.9	0.161
Feed conversion ratio								
D 1–14	1.07	0.97	1.09	1.14	1.22	1.23	0.11	0.116
D 15–28	1.38	1.30	1.53	1.43	1.43	1.39	0.09	0.574
D 1–28	1.31	1.23	1.43	1.38	1.38	1.37	0.09	0.463
Average water intake (l)								
D 1–14	1.09	1.24	1.14	1.08	1.04	1.13	0.03	0.448
D 15–28	2.68	2.69	2.74	2.51	2.49	2.94	0.08	0.527
D 1–28	3.75	3.90	3.86	3.62	3.53	4.05	0.07	0.232

^1^ Treatments include—(1) Negative Control treatment- chicks fed a basal corn-soybean meal-wheat–based diet; (2) In-feed antibiotics- chicks fed NC + 0.05% bacitracin methylene disalicylate and (3) In-water essential oil- chicks supplied the essential oil via the water route at the recommended dosage of 250 mL/1000 L of drinking water; (4) In ovo saline treatment- eggs injected with 0.2 mL of physiological saline (0.9% NaCl); (5) In ovo essential oil treatment- eggs injected with 0.2 mL of a saline + essential oil blend mixture at a dilution ratio of 2:1, (6) In ovo + in-water essential oil treatment- chicks offered the essential oil blend via the in ovo and in water route, successively. ^2^ SEM = Standard error of means. ^3^ Means within a row with different superscripts ^a,b,c^ differ (*p* < 0.05).

**Table 4:** Effect of essential oil delivery route on the relative weight of broiler chicken organs and serum immunoglobulins levels.

Parameters	Treatments ^1^						SEM ^2^	*p* Value
	Negative Control	In-Feed Antibiotics	In-Water Essential Oil	In Ovo Saline	In Ovo Essential Oil	In Ovo Essential Oil + In-Water Essential oil		
Bursa weight (g/Kg BW)	1.84	2.03	1.79	1.80	1.91	1.72	0.07	0.826
Liver weight (g/Kg BW)	27.5	27.5	29.3	30.0	29.2	31.8	0.51	0.066
Immunoglobulin G (Mg/mL)	0.74	0.44	0.78	1.29	0.76	0.68	0.31	0.189
Immunoglobulin M (Mg/mL)	0.11	0.07	0.12	0.15	0.11	0.08	0.02	0.289

^1^ Treatments include—(1) Negative Control treatment- chicks fed a basal corn-soybean meal-wheat–based diet; (2) In-feed antibiotics- chicks fed NC + 0.05% bacitracin methylene disalicylate and (3) In-water essential oil- chicks supplied the essential oil via the water route at the recommended dosage of 250 mL/1000 L of drinking water; (4) In ovo saline treatment- eggs injected with 0.2 mL of physiological saline (0.9% NaCl); (5) In ovo essential oil treatment- eggs injected with 0.2 mL of a saline + essential oil blend mixture at a dilution ratio of 2:1, (6) In ovo + in-water essential oil treatment- chicks offered the essential oil blend via the in ovo and in water route, successively. ^2^ SEM = Standard error of means.

**Table 5:** Effect of essential oil delivery route on plasma biochemical characteristics in broiler chickens.

Parameters	Treatments ^1^						SEM ^2^	*p* Value ^3^
	Negative Control	In-Feed Antibiotics	In-Water Essential Oil	In Ovo Saline	In Ovo Essential Oil	In Ovo Essential Oil + In-Water Essential Oil		
Electrolytes minerals (mmol·L^−1^)								
Sodium	150	142	143	145	145	151	1.96	0.561
Potassium	5.93	5.79	6.12	6.66	5.90	6.36	0.22	0.497
Sodium: Potassium	25.9	24.8	23.8	23.0	24.9	24.0	0.61	0.556
Chloride	110	105	106	106	107	112	1.28	0.565
Calcium	2.68	3.01	2.69	2.65	2.67	3.04	0.05	0.072
Phosphorus	1.78	1.66	2.01	1.92	1.73	1.78	0.07	0.690
Magnesium	0.87	0.86	0.78	0.82	0.77	0.84	0.03	0.519
Metabolites (mmol·L^−1^)								
Urea	0.29	0.30	0.26	0.31	0.22	0.32	0.02	0.352
Glucose	14.1	13.4	13.5	13.6	14.0	14.1	0.22	0.809
Cholesterol	3.34	2.91	2.72	3.08	3.18	3.42	0.09	0.233
Iron	15.8	14.1	14.4	16.6	15.1	17.9	0.71	0.589
Bile acids	15.7	17.7	13.8	14.0	18.8	19.2	3.79	0.393
Uric acid	328	327	342	383	364	396	16.3	0.622
Enzymes (U·L^−1^)								
Amylase	459	405	376	657	572	626	39.6	0.221
Alkaline phosphatase	4506	2614	4263	4879	4055	5157	398	0.173
Creatine kinase	4873 ^a,b^	7903 ^a^	3100 ^a,b^	2541 ^b^	4309 ^a,b^	2408 ^b^	609	0.022
Aspartate Aminotransferase	180 ^a^	186 ^a^	144 ^b^	143 ^b^	165 ^a,b^	138 ^b^	5.33	0.028
Gamma-Glutamyl Transferase	9.7	9.5	10.0	10.0	9.3	9.9	0.38	0.993
Lipase	24.5	22.2	21.4	21.5	19.5	27.7	1.46	0.661
Proteins (g·L^−1^)								
Total Proteins	28.1	23.7	24.1	25.9	24.7	27.8	0.80	0.336
Albumin	12.6	11.0	10.5	11.3	11.4	11.8	0.28	0.459
Globulin	15.7	12.9	13.5	14.5	13.5	16.0	0.58	0.268
Albumin: Globulin	0.76	0.84	0.78	0.78	0.83	0.75	0.02	0.547

^1^ Treatments include—(1) Negative Control treatment- chicks fed a basal corn-soybean meal-wheat–based diet; (2) In-feed antibiotics- chicks fed NC + 0.05% bacitracin methylene disalicylate and (3) In-water essential oil- chicks supplied the essential oil via the water route at the recommended dosage of 250 mL/1000 L of drinking water; (4) In ovo saline treatment- eggs injected with 0.2 mL of physiological saline (0.9% NaCl); (5) In ovo essential oil treatment- eggs injected with 0.2 mL of a saline + essential oil blend mixture at a dilution ratio of 2:1, (6) In ovo + in-water essential oil treatment- chicks offered the essential oil blend via the in ovo and in water route, successively. ^2^ SEM = Standard error of means. ^3^ Means within a row with different superscripts ^a,b^ differ (*p* < 0.05).

**Table 6:** Effect of essential oil delivery route on broiler chicken intestinal morphology.

Parameters		Treatments ^1^						SEM ^2^	*p* Value ^3^
	Intestinal Segment (Measured in mm)	Negative Control	In-Feed Antibiotics	In-Water Essential Oil	In Ovo Saline	In Ovo Essential Oil	In Ovo Essential Oil + In-Water Essential Oil		
Duodenum									
Villus Height		1.56 ^b,c^	1.54 ^b,c^	1.53 ^b,c^	1.50 ^c^	1.63 ^a^	1.59 ^a,b^	0.01	<0.001
Villus width		0.15 ^a,b^	0.15 ^a,b^	0.15 ^a,b^	0.14 ^b^	0.17 ^a^	0.17 ^a,b^	0.00	0.010
Crypt depth		0.14	0.14	0.13	0.13	0.14	0.14	0.00	0.29
Villus height: Crypt depth		10.96	10.66	11.69	10.90	11.48	11.64	0.18	0.307
Total mucosa thickness		1.70 ^a,b,c^	1.68 ^b,c^	1.66 ^b,c^	1.63 ^c^	1.78 ^a^	1.73 ^a,b^	0.01	<0.001
Jejunum									
Villus Height		0.90	0.88	0.89	0.87	0.93	0.92	0.01	0.192
Villus width		0.16	0.16	0.16	0.16	0.15	0.16	0.00	0.825
Crypt depth		0.13	0.13	0.13	0.13	0.13	0.14	0.00	0.431
Villus height: Crypt depth		6.47	6.19	6.75	6.25	7.16	6.51	0.14	0.126
Total mucosa thickness		1.05	1.03	1.02	1.02	1.07	1.07	0.01	0.240
Ileum									
Villus Height		0.48 ^a^	0.45 ^a,b^	0.43 ^b^	0.45 ^a,b^	0.49 ^a^	0.46 ^a,b^	0.01	<0.001
Villus width		0.15	0.16	0.16	0.15	0.16	0.16	0.00	0.756
Crypt depth		0.10	0.11	0.09	0.10	0.10	0.10	0.38	0.135
Villus height: Crypt depth		4.91	4.24	4.72	4.77	4.84	4.80	0.09	0.147
Total mucosa thickness		0.58 ^a,b^	0.57 ^a,b^	0.52 ^c^	0.54 ^b,c^	0.60 ^a^	0.56 ^a,b,c^	0.38	<0.001

^1^ Treatments include—(1) Negative control treatment- chicks fed a basal corn-soybean meal-wheat–based diet; (2) In-feed antibiotics- chicks fed NC + 0.05% bacitracin methylene disalicylate and (3) In-water essential oil- chicks supplied the essential oil via the water route at the recommended dosage of 250 mL/1000 L of drinking water; (4) In ovo saline treatment- eggs injected with 0.2 mL of physiological saline (0.9% NaCl); (5) In ovo essential oil treatment- eggs injected with 0.2 mL of a saline + essential oil blend mixture at a dilution ratio of 2:1, (6) In ovo + in-water essential oil treatment- chicks offered the essential oil blend via the in ovo and in water route, successively. ^2^ SEM = Standard error of means. ^3^ Means within a row with different superscripts ^a,b,c^ differ (*p* < 0.05).

## Data Availability

The data presented in this study are available on request from the corresponding author.

## Supplementary Materials

The following are available online at https://www.mdpi.com/article/10.3390/ani11123386/s1, Table S1: Effect of essential oil delivery route on hatch performance and chick quality at Pretrial.
