# Supplementary material for: Essential Oil Delivery Route: Effect on Broiler Chicken’s Growth Performance, Blood Biochemistry, Intestinal Morphology, Immune, and Antioxidant Status
Source: Animals (Basel). 2021 Nov 26;11(12):3386. doi: 10.3390/ani11123386 (PMC8697888; doi:10.3390/ani11123386)
Supplement: Supplementary file 1 [file animals-11-03386-s001.zip › animals-1475389-supplementary.pdf]

animals

MDPI

Supplementary Material

Table S1. Effect of essential oil delivery route on hatch performance and chick quality at pretrial.

Hatch Parameters	Treatments [1]				SEM [2]	*p* Value
	Non-Injected	*In ovo* Saline	*In ovo* Essential oil	*In ovo* Essential oil + Saline		
Hatchability (%)	100 [a]	81.5 [ab]	79.5 [b]	74.6 [b]	3.18	0.008
Average Chick Weight (g)	42.1 [ab]	35.1 [bc]	31.7 [c]	43.8 [a]	1.52	<0.001
Average Chick Length (cm)	19.7	18.6	18.7	19.2	0.20	0.216

[1] Treatments include — 1) non-injected eggs; 2) *in ovo* Saline group- injected with 0.2 mL of physiological saline (0.9% NaCl); 3) *in ovo* essential oil group- injected with 0.2 mL essential oil blend mixture containing phytonutrients star anise, cinnamon, rosemary, and thyme oil; 4) *in ovo* essential oil + saline group- injected with 0.2 mL essential oil blend mixture (containing phytonutrients star anise, cinnamon, rosemary, and thyme oil) and saline (0.9% NaCl) solution at a dilution ratio of 1:1. Each treatment groups had 60 eggs each (average weight 78.4 ± 2.73 g; mean ± SE), sourced from Ross 308 broiler breeders. [2] SEM = Standard error of means. [a,b,c] Means within a row with different superscripts differ ($p < 0.05$).
